# Circulating Tumor-Derived Endothelial Cells: An Effective Biomarker for Breast Cancer Screening and Prognosis Prediction

**DOI:** 10.1155/2022/5247423

**Published:** 2022-08-28

**Authors:** Tuo Han, Juanjuan Zhang, Dong Xiao, Binhui Yang, Liang Chen, Chao Zhai, Feifei Ding, Yue Xu, Xiaoyu Zhao, Jiangman Zhao

**Affiliations:** ^1^Department of Oncological Surgery, 3201 Hospital Affiliated to Xi'an Jiaotong University School of Medicine, Hanzhong 723000, Shaanxi, China; ^2^Department of Surgical Oncology, Quanzhou First Hospital Affiliated to Fujian Medical University, Quanzhou 362000, China; ^3^Shanghai Biotecan Pharmaceuticals Co. Ltd, Shanghai Zhangjiang Institute of Medical Innovation, Shanghai 201204, China

## Abstract

**Background:**

Circulating tumor-derived endothelial cell (CTEC) is a new potential tumor biomarker to be associated with cancer development and treatment efficacy. However, few evidences are available for breast cancer.

**Methods:**

Eighty-nine breast cancer patients were recruited, and preoperative and postoperative blood samples were collected. Besides, 20 noncancer persons were enrolled as controls. An improved subtraction enrichment and immunostaining-fluorescence in situ hybridization (SE-iFISH) method was adopted to codetect CD31+ aneuploid CTEC and CD31− aneuploid circulating tumor cell (CTC). Then, the clinical significance of CTCs and CTECs on breast cancer screening and prognosis prediction was evaluated and compared.

**Results:**

The positive rate of CTCs and CTECs in newly diagnosed breast cancer patients was 68.75% and 71.88%. Among detected aneuploid circulating rare cells, CTEC accounts for a greater proportion than CTC in breast cancer patients. CTEC-positive rate and level were significantly higher in breast cancer patients with lymph node metastasis (LNM) than those without LNM (*P*=0.043), while there was no significant difference in CTC. CTEC (area under the curve, AUC = 0.859) had better performance than CTC (AUC = 0.795) to distinguish breast cancer patients from controls by receiver operator characteristic curve analysis. Preoperative CTEC count ≥ 2 was a significant risk factor for reducing PFS of breast cancer patients.

**Conclusions:**

CTECs may function as a reliable supplementary biomarker in breast cancer screening and prognosis prediction.

## 1. Introduction

Breast cancer is the most common cancer in women worldwide [1]. About 20–30% of breast cancers have developed locally advanced or metastasis, which results in poor prognosis [2]. Postoperative recurrence and metastasis are the major causes of mortality in breast cancer patients [3]. Because of its complexity, the traditional methods of cancer detection are limited to comprehensively capturing the characteristics of breast tumors. Hence, there are still several limitations of breast cancer management including early diagnosis, prediction of relapse and prognosis, and monitoring response to treatment.

As a liquid biopsy method, circulating tumor cell (CTC) is a commonly used biomarker for breast cancer diagnosis and prognosis prediction. Previous studies have shown that CTCs numbers in peripheral blood were directly associated with the prognosis of breast cancer patients [4–8]. Persistently high CTCs numbers in patients always indicate significantly increased risks for relapse and postoperative micrometastasis [9, 10]. The presence of CTCs after neoadjuvant chemotherapy was also found to be relevant to early metastatic relapse and worse disease-free survival [11]. CTCs have also been used for therapeutic evaluation, in which patients with persistent CTCs after completion of (neo) adjuvant chemotherapy have an increased risk of relapse [12–14]. For patients receiving palliative chemotherapy, CTCs numbers after the first cycle of treatment showed strong relevance to the therapeutic response [15].

The technical approaches of enrichment and identification were multifarious, mainly based on polyploidy [16, 17], CK positivity, and EpCAM positive [18–21], which always confused circulating tumor-derived endothelial cells (CTECs) with CTCs. CTECs are tumor-derived endothelial cells shed into the peripheral circulation of patients [22, 23]. Peter Ping Lin et al. developed an improved subtraction enrichment and immunostaining-fluorescence in situ hybridization (SE-iFISH) method, which could codetect aneuploid CTC and CTEC [22]. Increased CTECs counts were found to be a high-risk factor for poor outcomes in nonsmall cell lung cancer (NSCLC) patients [24]. CTECs quantification is also a promising tool for treatment monitoring for neoadjuvant letrozole plus low-dose cyclophosphamide therapy in estrogen receptor-positive breast cancer [25]. However, there are few studies comprehensively expounding the characteristics of CTECs and CTCs and their clinical significance during breast cancer diagnosis and treatment.

In this study, we applied the SE-iFISH method developed by Cytelligen (San Diego, CA, USA) to codetect aneuploid CTC and CTEC [22]. A total of 89 breast cancer patients and 20 noncancer controls were recruited, and peripheral blood was collected to detect CTCs and CTECs. Cytogenetic characteristics of CTCs and CTECs during the process of diagnosis and treatment and their correlation with clinical and pathological factors were comprehensively analyzed. The clinical significance of CTCs and CTECs on breast cancer screening and prognosis prediction was also evaluated and compared.

## 2. Materials and Methods

### 2.1. Participants and Sample Collection

A total of 89 female breast cancer patients and 20 noncancer controls were recruited from 3201 Hospital Affiliated to Xi'an Jiaotong University School of Medicine and Quanzhou First Hospital Affiliated to Fujian Medical University between April 2018 and April 2020. The clinical characteristics of 89 breast cancer patients are given in Table 1. Seventy-five patients received surgery following adjuvant therapy, and 14 patients underwent neoadjuvant chemotherapy (NCT) following surgery and adjuvant therapy (Table 1).

**Figure 1:**
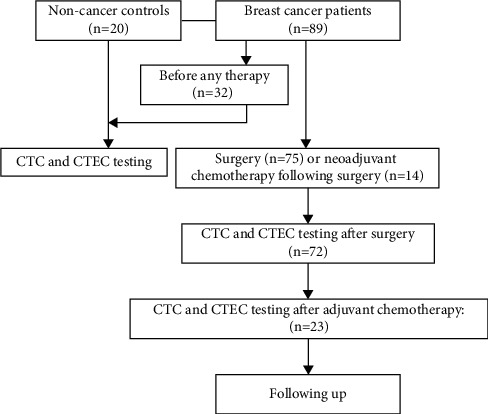
Flowchart. A total of 89 breast cancer patients were enrolled.

In order to detect CTCs and CTECs, 7.5 mL of peripheral blood was collected by acid citrate dextrose (ACD) anticoagulant tube (Becton Dickinson, Franklin Lakes, NJ, USA) from 20 noncancer controls and 32 breast cancer patients. There are 72 patients after surgery who received CTCs and CTECs tests, while 23 of them received twice, after surgery and postoperative adjuvant chemotherapy. The design of this study is shown in Figure 1.

This study involves human participants and was approved by the Ethical Committee of Department of Oncological Surgery, 3201 Hospital Affiliated to Xi'an Jiaotong University and Quanzhou First Hospital Affiliated to Fujian Medical University. The experiments were conducted after collecting the informed consent of each subject, and the study conforms with The Code of Ethics of the World Medical Association (Declaration of Helsinki) in the British Medical Journal (18 July 1964).

### 2.2. Subtraction Enrichment (SE)

CTCs and CTECs were enriched with a Human Circulating Rare Cell Subtraction Enrichment kit (Cytelligen, San Diego, CA, USA) according to the manufacturer's instructions and previous studies' suggestions [22]. In brief, 7.5 mL of peripheral blood was centrifuged at 800 × *g* for 8 min at room temperature to remove plasma. Then, the lower layer of cells was transferred into centrifuge tubes containing 3 mL of hCTC separation matrix, and red blood cells were discarded by centrifugation at 450 × *g* for 8 min at room temperature. Buffy coat cells were collected in new tubes and then incubated with an immunomagnetic particle-conjugatedanti-CD45 antibody for 20 min with gentle shaking. WBCs bound to immunobeads were depleted using a magnetic separator. The solution without beads was centrifuged at 450 × *g* for 8 min following rinsed twice by hCTC buffer at room temperature. Finally, the cell pellet was completely mixed with cell fixative, and the mixture was coated on glass slides and dried overnight at 30°C, which would be identified by immunostaining-fluorescence in situ hybridization (iFISH).

### 2.3. Immunostaining-Fluorescence In Situ Hybridization (iFISH)

For CTCs and CTECs identification, the dried monolayer cells on the coated and formatted CTC slides were rinsed and incubated with saline-sodium citrate buffer for 10 min following dehydration in ethanol for 2 min. Centromere probe 8 (CEP8) spectrum orange (Cytelligen, San Diego, CA, USA) was added to the CTC slides, which were denatured at 76°C for 10 min and hybridized for 4 h at 37°C. Then, the hybridization slides were subsequently darkly incubated with AlexaFluor® 594-conjugatedanti-CD45 IgG (spectrum red) and AlexaFluor® 488-conjugatedanti-CD31 IgG (spectrum green) for 2 h at room temperature. Finally, DAPI (spectrum blue) was added to the CTC slides and was subjected to fluorescence microscope scanning and analyses.

### 2.4. CTC and CTEC Identification

**Figure 2:**
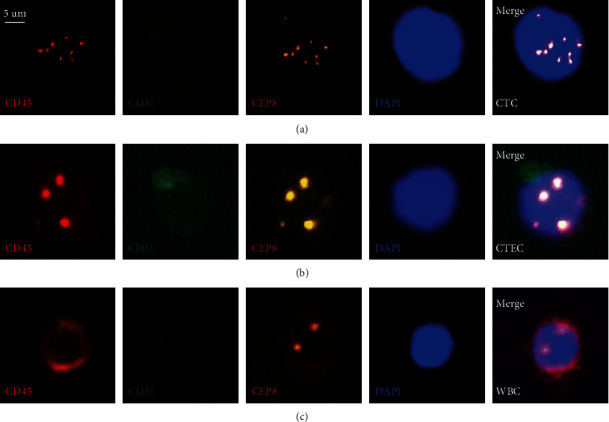
Identification of CTC, CTEC, and WBC by SE-iFISH. (a) CTC (CD31−/CD45−/DAPI+/CEP8 > 2); (b) CTEC (CD31+/CD45−/DAPI+/CEP8 > 2); (c) WBC (CD31−/CD45+/DAPI+/CEP8 = 2). Scale bars, 5 *μ*m; CD45, red; CD31, green; CEP8, orange; DAPI, blue.

The CTC were confirmed by CD31−/CD45−/DAPI+/CEP8 > 2 (Figure 2(a)), and the CTEC was defined as CD31+/CD45−/DAPI+/CEP8 > 2 (Figure 2(b)). The interference by leukocyte should be excluded using CD45+ (Figure 2(c)) [26], which was defined as CD31−/CD45+/DAPI+/CEP8 = 2.

### 2.5. Statistical Analysis

Statistical analysis and graphical plots were performed using SPSS 22 (IBM Corp.), GraphPad Prism 6 (La Jolla, CA, USA), and R project. The differences of categorical variables in distribution among groups were analyzed using the chi-square test or the Fisher exact test. Differences of continuous variables with normal distribution among groups were compared by the *t*-test, while nonparametric tests (Mann–Whitney *U* test or Kruskal–Wallis H test) were used if they are not consistent with normal distribution. Receiver-operating characteristic (ROC) curve was used to assess the diagnostic efficacy of variables between controls and patients. Kaplan–Meier survival curves and log-rank tests were used to compare the differences in progression-free survival (PFS) rate between the two groups. Cox proportional hazards regression analyses were carried out to identify risk factors of poor prognosis. *P* < 0.05 was considered to indicate a statistically significant difference.

## 3. Results

### 3.1. Analysis of CTCs and CTECs in Breast Cancer Patients before Any Therapy

**Figure 3:**
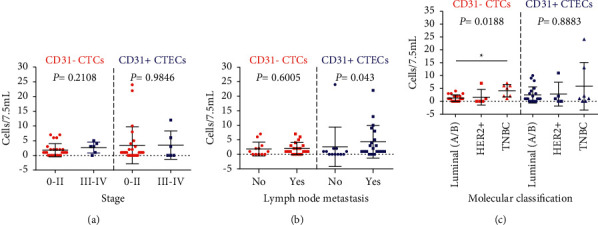
CD31− CTC and CD31+ CTEC in newly diagnosed breast cancer patients. Comparison of CTC and CTEC abundance according to tumor stage (a), lymph node metastasis (b), and molecular classification (c).

Table 2 provides the clinical characteristics and the CTC test results of 32 patients who received CTC and CTEC tests before any therapy when newly diagnosed. In addition, we also compared the levels of CTCs and CTEC according to the stage, lymph node metastasis (LNM) status, and molecular classifications (Figure 3). We found that CTEC-positive rate (Table 2, *P*=0.033) and level (Figure 3(b), *P*=0.043) were significantly higher in breast cancer patients with LNM than those without LNM, while there was no significant difference in CTC-positive rate (Table 2, *P*=0.325) and level (Figure 3(b), *P*=0.6005). CTC was detected in all triple-negative breast cancer (TNBC) patients (100%, 7/7), and its positive rate was much higher than that in HER2+ (40%, 2/5) and luminal A/B (65%, 13/20) classification.  Figure 3(c) also show that TNBC patients had a higher level of CTC than other classifications (*P*=0.0188). However, there was no significant difference in the level of CTC and CTEC between stage 0–II and III-IV patients. A novelty of the results was that elder patients (>50 years) had a significantly higher CTEC-positive rate than younger patients (*P*=0.050) and overweight patients (BM ≥ 24.0) had a higher CTC-positive rate (*P*=0.024) than other patients (BMI < 24.0).

### 3.2. Characteristics of CTCs and CTECs in Breast Cancer Patients

**Figure 4:**
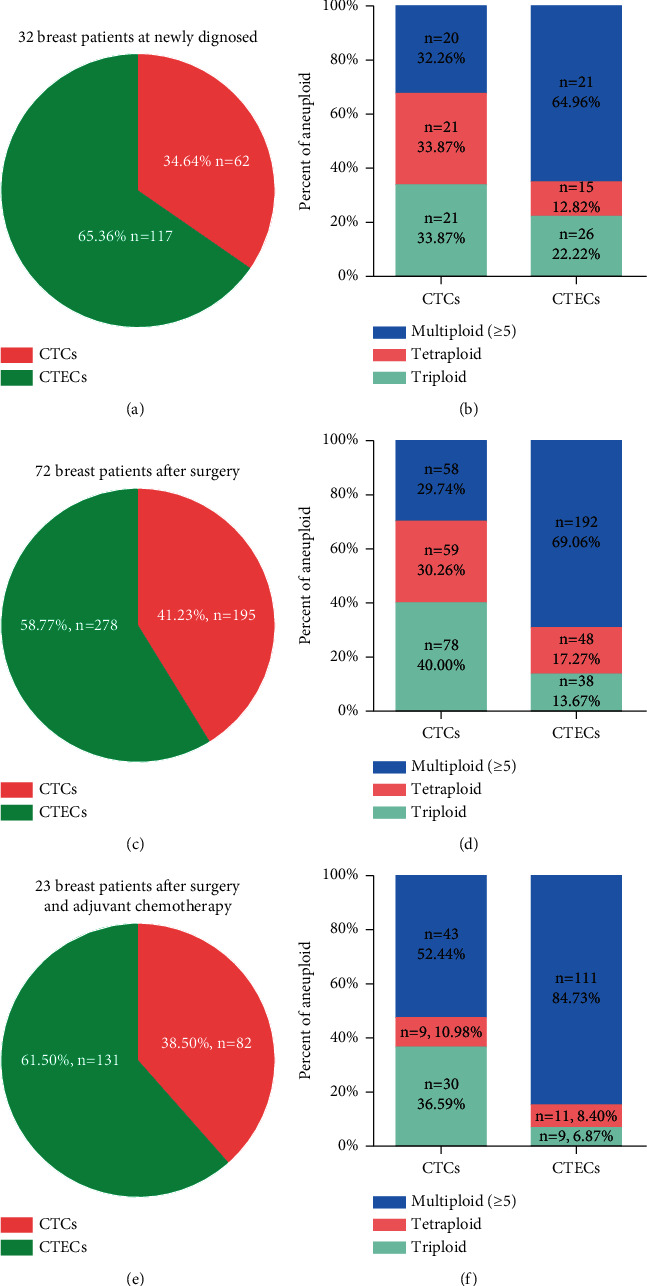
Distribution of CTCs and CTECs results according to ploidy of breast cancer patients. (a)-(b) 32 breast cancer patients when newly diagnosed before any therapy; (c)-(d) 72 breast patients after surgery; (e)-(f) 23 patients after surgery and adjuvant chemotherapy.

Figure 4 shows the distribution of CTC and CTEC and their ploidy ratio. The results showed that CTEC accounts for a greater proportion than CTC in blood of breast cancer patients (Figures 4(a), 4(c), and 4(e)). In 32 newly diagnosed breast patients, 62 CTCs (34.64%) and 117 CTECs (65.36%) were detected (Figure 4(a)). The CTC and CTEC proportions of patients after surgery (Figure 2(c)) and adjuvant chemotherapy (Figure 3(e)) were similar to the patients before any therapy. During the first two times of tests before adjuvant chemotherapy, the ratio of triploid, tetraploid, and multiploidy of CTCs was approximately equal (Figures 4(b), 4(d)), while multiploidy (≥5) accounted for the majority of CTECs (Figures 4(b), 4(d)). We found that the multiploidy (≥5) proportion of CTC and CTEC had obviously increased to 52.44% and 84.73%, respectively, after adjuvant chemotherapy (Figure 4(f)).

**Figure 5:**
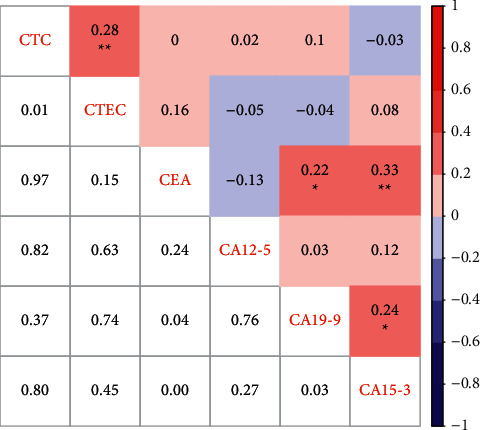
Heatmap of correlation among CTC, CTEC, CEA, CA12-5, CA19-9, and CA15-3. The value in the grids of the upper triangle is the Spearman correlation coefficient (Spearman *r*), which is marked by colors (red, positive correlation; blue, negative correlation). The value in grids of the lower triangle is *P* value of Spearman correlation. ^*∗*^*P* < 0.05, ^*∗∗*^*P* < 0.01, and ^*∗∗∗*^*P* < 0.001.

To comprehensively understand the relationship and difference between novel and traditional cancer biomarkers, we calculated Spearman's rank correlation coefficient of CTC, CTEC, CEA, CA12-5, CA19-9, and CA15-3 (Figure 5). CTC was significantly and positively correlated with CTEC (*r* = 0.28, *P*=0.01). Meanwhile, CEA had markedly positive correlations with CA19-9 (*r* = 0.22, *P*=0.04) and CA15-3 (*r* = 0.33, *P* < 0.01), and CA19-9 was positively correlated with CA15-3 (*r* = 0.24, *P*=0.03). No significant correlation was found between novel biomarkers (CTC and CTEC) and traditional biomarkers (CEA, CA12-5, CA19-9, and CA15-3). These results indicated CTC and CTEC were independent of traditional cancer biomarkers.

### 3.3. Clinical Screening Value of CTC and CTEC in Breast Cancer Patients

**Figure 6:**
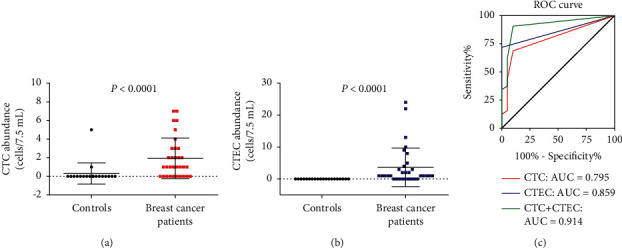
Comparison of CTCs (a) and CTECs (b) abundance between controls and breast cancer patients. (c) The ROC curve of CTCs, CTECs, and their sum (CTCs + CTECs) to distinguish breast cancer patients from controls.

To evaluate the clinical application value of CTC and CTEC for breast cancer screening, 20 noncancer participants were recruited to detect CTC and CTEC as controls. CTC was not detected in 18 of 20 (90%) controls, and all the 20 controls' CTEC (100%) was negative. For breast cancer patients, the positive rate of CTC and CTEC was 68.75% (22/32) and 71.88% (23/32), respectively. The mean value of CTC and CTEC was significantly higher in breast cancer patients than those in controls (Figures 6(a) and 6(b), *P* < 0.0001). Results of ROC curve analysis showed that CTEC (AUC = 0.859) had better performance than CTC (AUC = 0.795) for breast cancer screening. After summing the aneuploid cells (CTC plus CTEC), the AUC was widely promoted to 0.914. This result suggested aneuploid cells from peripheral blood were an ideal biomarker for breast cancer screening.

### 3.4. Clinical Prognostic Significance of CTCs and CTECs in Breast Cancer Patients

**Figure 7:**
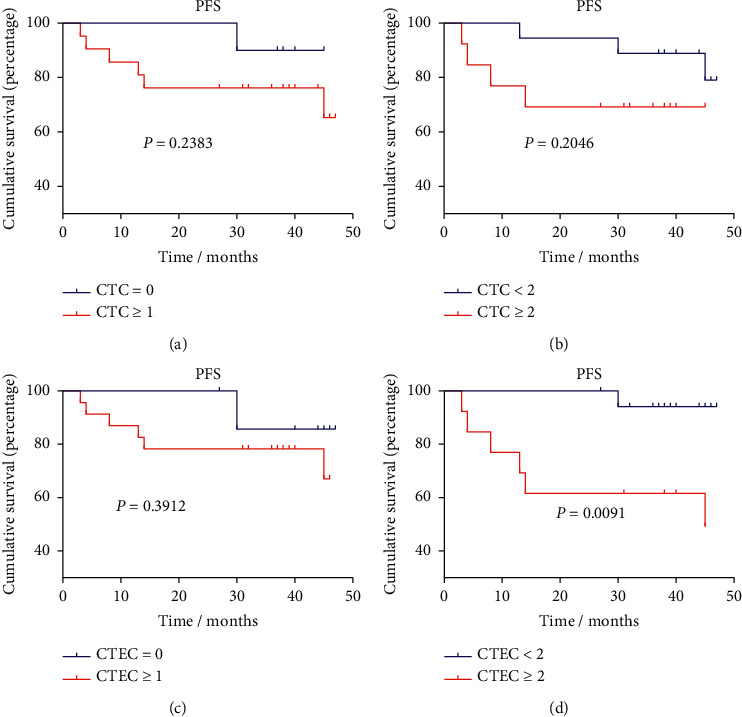
Kaplan–Meier survival curve analysis of PFS in breast cancer patients according to CTC and CTEC counts before any therapy. (a) CTC = 0 vs. CTC ≥ 1; (b) CTC < 2 vs. CTC ≥ 2; (c) CTEC = 0 vs. CTEC ≥ 1; (d) CTEC < 2 vs. CTEC ≥ 2.

**Figure 8:**
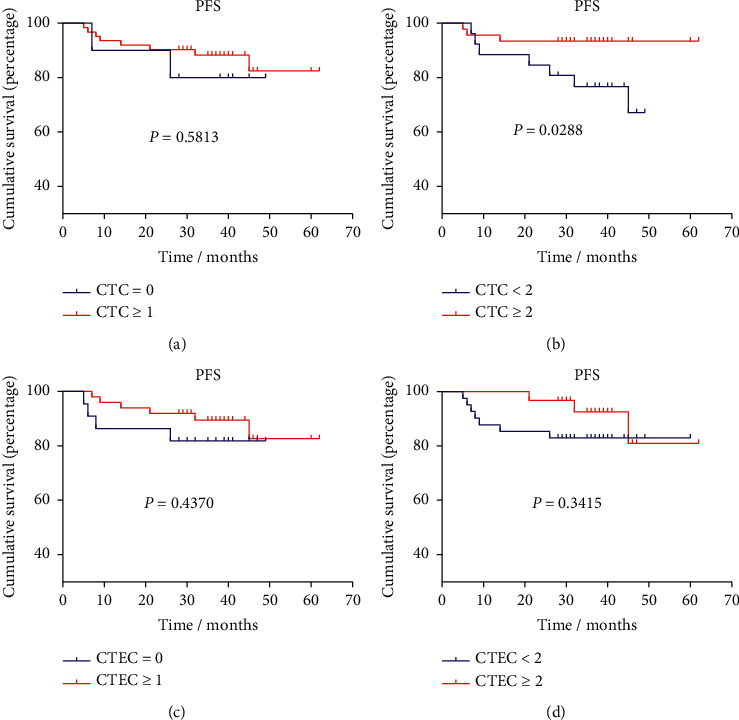
Kaplan–Meier survival curve analysis of PFS in breast cancer patients according to CTC and CTEC counts after surgery. (a) CTC = 0 vs. CTC ≥ 1; (b) CTC<2 vs. CTC ≥ 2; (c) CTEC = 0 vs. CTEC ≥ 1; (d) CTEC < 2 vs. CTEC ≥ 2.

All the patients were followed-up until March 2022, using progression (recurrence or metastasis) as the end of follow-up. The median follow-up time was 37 months (3–62 months). During the follow-up period, 14 patients had recurrence, 1 patient was lost of follow-up, and 74 patients were progression-free. We separately plotted the progression-free survival curves according to the CTC and CTEC values before and after the surgical operation. Preoperative CTEC count ≥2 was a significant risk factor for reducing the PFS of breast cancer patients (*P*=0.0091, Figure 7(d)). However, preoperative CTC enumeration showed no significant impact on PFS (*P* > 0.05) and postoperative quantity of CTEC also had no significant influence on PFS (Figure 8). Surprisingly, the increased postoperative CTC count (≥2 cells/7.5 mL) can even predict a better prognosis (Figure 8).

## 4. Discussion

The tumor microenvironment (TME) is a complex system comprised of cancer cells and their surrounding cells such as endothelial cells, cancer-associated fibroblasts, immune cells, and so on [27]. CTCs were cancer cells shed into peripheral blood, and similarly, CTECs were the tumor-derived endothelial cells disseminated to the blood circulation [28]. In the last decade, numerous studies have verified that the CTCs, as the main biomarker in liquid biopsy, have been used for screening and monitoring treatment efficacy, as well as predicting the prognosis of many cancers [18–21]. CTECs mostly harbor mixed properties of both endothelial vascularization and cancerous malignancy [28]. In most studies, the CTC detecting methods only identify CTC using the following markers such as cellular size, EpCAM and CK expression, and aneuploid chromosomes, which cannot distinguish CTECs from CTCs.

In this study, we used an improved SE-iFISH method, which could codetect and distinguish aneuploid CTEC by adding an anti-CD31 antibody from aneuploid CTC. Then, 89 breast cancer patients and 20 noncancer controls were enrolled to receive CTC and CTEC tests of peripheral blood. First, we exhibited the cytogenetic characteristics of CTCs and CTECs during the course of breast cancer. We found that higher CTECs quantity and positive rate were significantly correlated with LNM of breast cancer, but no significant difference was found in CTC level. Since endothelial cells make up the lining of the tumor vasculature and lymphogenous cancer metastases are mainly impacted by lymphatic vessel-related lymphangiogenesis [28], an active cross-talk between blood and lymphatic vessel endothelial cells in the TME had been thought to impact cancer cells' trend to lymphogenous or hematogenous metastasis pathway [29]. Hence, our results provided evidence for CTECs' relevance to the process of tumor lymphangiogenesis, which approved that an increased CTEC count gave a reminder of LNM. In addition, our results showed the level of CTECs was higher than the level of CTCs in breast cancer patients. Inversely, other studies reported that CTCs occupied the majority of circulating aneuploid cells in colorectal cancer [26] and nonsmall cell lung cancer [30]. A potential mechanism is that epithelial-mesenchymal transition (EMT) and endothelial-to-mesenchymal transition (EndoMT) are generally recognized to be related to cancer cell metastasis, which may proceed diversely in different cancers due to diverse characteristics of the respective blood and lymphatic vessel systems [29].

In fact, many studies have shown that CTECs are associated with the development of cancers. CTECs counts were found to be increased in nonsmall cell lung cancer (NSCLC) patients compared with controls and were considered as the high-risk factor of recurrence [24]. CTECs have previously been verified to be effective biomarkers for colorectal cancer screening [26]. The previous studies of CTECs in breast cancer mostly focused on monitoring treatment efficacy [31, 32], especially antivascular endothelial growth factor-A (anti-VEGF-A) antibody bevacizumab [33–35]. Only a few studies reported its clinical value on breast cancer screening. In this study, we examined the potential of CTC and CTEC for breast cancer screening using a small size of sample involving 20 controls and 32 newly diagnosed breast cancer patients. The results showed the capability of CTEC (AUC = 0.859) was superior to CTC (AUC = 0.795), and the combination of aneuploid CD31+ CTEC and CD31− CTC exhibited the best performance (AUC = 0.914). Especially, CTEC has 100% of specificity in controls. The origin of tumor-derived endothelial cells is formed based on two processes: cancerization of stromal cells and endothelialization of cancer cells. In addition, it is considered that endothelialization of malignant cancer cells may constitute the primary pathway for the formation of tumor-derived endothelial cells [36]. Increasing evidence suggests that cancer results from altered organ homeostasis rather than deregulated control of a single cell or a group of cells [37]. This may give theoretical support for our results on why CTEC has no false-positive in noncancer controls. Comprehensive consideration of CTC and CTEC status may be the optimal strategy for breast cancer screening or diagnosis, which needs to be validated in larger size cohorts in future studies.

In this study, we found surgery had no obvious effect on the heteroploid distribution of CTCs and CTECs. However, preoperative and postoperative CTC/CTEC count had different influences on PFS of breast cancer. Preoperative CTEC count equal to or greater than 2 cells/7.5 mL was the risk factor of shorter PFS of breast cancer, while no similar phenomena appeared on postoperative CTC or CTEC number. Previous studies have proved that the level of CTC or CTEC will rise within a short period when patients were receiving treatment such as surgery or chemotherapy [31, 38]. Ma et al. reported the aneuploid CTECs in peripheral blood of locally advanced breast cancer patients initially increased and then decreased during neoadjuvant chemotherapy [31]. Therefore, it is not suggested to detect CTC and CTEC during or shortly after treatment at a single time point to assess the recurrent risk of breast cancer. Increased preoperative CTEC is a potential risk factor for recurrence or distant metastasis, and it may provide instruction on the selection of operational ways to reduce recurrent risk, in advance.

There are a few limitations to this study. First, the findings are limited because of the small size of breast cancer patients and controls. Second, since the follow-up period is not long enough, less than half of the patients reached the follow-up endpoint. In addition, the clinical pathways of our patients were diverse, which may disturb the results. A prospective study with a larger size of the cohort and a longer follow-up period is necessary to validate the findings.

## 5. Conclusions

In summary, we used an improved SE-iFISH method to codetect CTC and CTEC in peripheral blood of 89 breast cancer patients and 20 controls. The CTEC level was positively correlated with CTC, and both of them were independent of traditional cancer biomarkers such as CA15-3, CA19-9, and CA12-5. CTECs are a more effective biomarker for breast cancer screening and prognosis prediction than CTCs.

## Figures and Tables

**Table 1:** Clinical characteristics of all 89 breast cancer patients in this study.

Clinical characteristics	Total	Proportion (%)
Age (years)		
≤50	46	51.69
>50	43	48.31

BMI		
<24.0	52	58.43
≥24.0	37	41.57

Molecular classification		
Luminal A	8	8.99
Luminal B	46	51.69
HER2+	16	17.98
TNBC	19	21.35

Stage		
0	2	2.25
I	12	13.48
II	53	59.55
III	19	21.35
IV	3	3.37

Lymph node metastasis		
Yes	52	58.43
No	37	41.57

Therapy		
Surgery following adjuvant therapy	75	84.27
NCT following surgery and adjuvant therapy	14	15.73

Stage 0, pTisN0M0.

**Table 2:** CTC and CTECs status according to clinical characteristics of newly diagnosed breast cancer patients before any therapy.

Clinical characteristics	Total	CTCs		*P* value	CTECs		*P* value
		Positive (*n* = 22)	Negative (*n* = 10)		Positive (*n* = 23)	Negative (*n* = 9)	
Age (years)				0.467			0.050^*∗*^
≤50	19	12	7		11	8	
>50	13	10	3		12	1	

BMI				0.024^*∗*^			0.704
<24.0	19	10	9		13	6	
≥24.0	13	12	1		10	3	

Molecular classification				0.073			0.905
Luminal (A/B)	20	13	7		14	6	
HER2+	5	2	3		4	1	
TNBC	7	7	0		5	2	

Stage				0.637			0.648
0–II	26	17	9		18	8	
III-IV	6	5	1		5	1	

Lymph node metastasis				0.325			0.033^*∗*^
Yes	20	15	5		17	3	
No	12	7	5		6	6	

^*∗*^ *P* < 0.05, ^*∗∗*^*P* < 0.01, ^*∗∗∗*^*P* < 0.001.

## Data Availability

The data used to support the findings of this study are included within the article.
